# A pilot investigation of e‐cigarette use and smoking behaviour among patients with chronic airway disease or respiratory symptoms

**DOI:** 10.1111/crj.13445

**Published:** 2021-09-13

**Authors:** Hye Seon Kang, Jae Woo Jung, Hye Jung Park, Dong Il Park, Jong Sook Park, Joo Hun Park, Sang Haak Lee, Eun Mi Chun, Jae Yeol Kim, Hye Sook Choi, Korean Smoking Cessation Study Group

**Affiliations:** ^1^ Division of Respiratory and Critical Care Medicine, Department of Internal Medicine, College of Medicine The Catholic University of Korea Seoul Republic of Korea; ^2^ Division of Respiratory and Critical Care Medicine, Department of Internal Medicine, College of Medicine Chung‐Ang University Seoul Republic of Korea; ^3^ Division of Respiratory and Critical Care Medicine, Department of Internal Medicine, College of Medicine Yonsei University Seoul Republic of Korea; ^4^ Division of Respiratory and Critical Care Medicine, Department of Internal Medicine, College of Medicine Chungnam National University Daejeon Republic of Korea; ^5^ Division of Respiratory and Critical Care Medicine, Department of Internal Medicine, College of Medicine Soonchunhyang University Bucheon Republic of Korea; ^6^ Division of Respiratory and Critical Care Medicine, Department of Internal Medicine, College of Medicine Ajou University Suwon Republic of Korea; ^7^ Division of Respiratory and Critical Care Medicine, Department of Internal Medicine, College of Medicine Ewha Womans University Seoul Republic of Korea; ^8^ Division of Respiratory and Critical Care Medicine, Department of Internal Medicine, College of Medicine Kyung Hee University Hospital Seoul Republic of Korea

**Keywords:** asthma, chronic obstructive lung disease, electronic nicotine delivery systems, nicotine dependence, respiratory symptoms

## Abstract

**Background:**

This pilot study aimed to investigate the current status of e‐cigarettes (ECs) use patterns among patients with chronic airway disease or chronic respiratory symptoms and the effects of ECs use on respiratory and mental health.

**Methods:**

A cross‐sectional survey was conducted at the outpatient clinic of eight teaching hospitals in South Korea between November 2019 and December 2019. All adult ECs users (19 years and above) who visited the outpatient clinic as a patient with chronic airway disease or chronic respiratory symptoms were eligible to participate in this study.

**Results:**

A total of 51 subjects responded to the survey. Most of the participants were male (92.2%) and the mean age was 41.8 years. Dominant airway diseases were asthma and chronic obstructive pulmonary disease. Most of the subjects had a history of cigarette smoking, and 19 subjects were dual users of current cigarettes and ECs. Most of the subjects started ECs use due to health‐related reasons. When comparing exclusive ECs users and dual users, St. George's respiratory questionnaire (SGRQ) scores, the proportion of cases with moderate to severe depressive symptoms, and average Fagerstrom test for nicotine dependence scores for ECs were higher in dual users than exclusive ECs users (mean 4.64 vs. 2.38, *p* = 0.006), respectively.

**Conclusion:**

Most of the subjects started ECs use due to health concerns, but dual users have more respiratory symptoms and higher nicotine dependence in this pilot study. One hypothesis that comes from these results is that greater nicotine dependence may influence behaviours, habits, and views about ECs. These preliminary observations need confirmation in a large cohort.

## 1 BACKGROUND

The first e‐cigarette (EC) was manufactured in 2003 by Hon Lik, a Chinese pharmacologist, with an intent to enable his father, who was a lung cancer patient and a heavy cigarette smoker, to quit smoking.^1^ Since the introduction of ECs into the Korean market, their prevalence has grown rapidly. After the introduction of IQOS in 2017, the market for cigarette type ECs has been estimated to be worth 1.67 billion (approximately 1.97 trillion won), and Korea is the worlds' second‐largest market after Japan.^2^

Even though a few studies have documented the usage trend of ECs in subjects with chronic airway disease, a recent study demonstrated an increase in the prevalence of ECs use from 5% to 12%–16% in chronic obstructive pulmonary disease (COPD) patients.^3^ Chronic airway disease is associated with cigarette smoking; ECs have been adopted by current conventional cigarettes (CCs) users as a safer alternative to deliver nicotine or a cessation device of CCs. However, the effects of ECs on CCs reduction and cessation are controversial.^4^, ^5^ A recent meta‐analysis demonstrated that ECs may interfere with the efforts imparted to quit cigarette smoking.^6^

Since the introduction of ECs, there has been an increase in the prevalence of ECs use and changes in the smoking pattern. The proportion of dual users (ECs and CCs) is constantly increasing in Korea.^7^ However, the current status of ECs use in patients with chronic airway disease in Korea is not well understood.

In our pilot study, we aimed to investigate the current status of ECs use patterns among patients with chronic airway disease or chronic respiratory symptoms and compare respiratory symptoms and mental health according to smoking behaviours including ECs use.

## 2 MATERIALS AND METHODS

### 2.1 Study population and data collection

This study was conducted between November 2019 and December 2019. A cross‐sectional survey was conducted at the outpatient clinic of eight teaching hospitals in South Korea. All the adult ECs users (19 years and above) of both the genders who visited the respiratory outpatient clinic as a patient with chronic airway disease or chronic respiratory symptoms were eligible to participate in this study. EC user was defined as who used ECs on some days or every day in the past 30 days. The subjects who declined to participate in the study were the ones with cognitive impairment and/or could not complete the questionnaire and hence were excluded.

A total of 51 subjects responded to the survey, which included a questionnaire concerning the status of ECs use, perception of ECs, the motivation of selecting ECs, nicotine dependence, respiratory symptoms, modified Medical Research Council Dyspnea scale, asthma control test, COPD assessment test (CAT), EuroQol‐5 dimension‐5 level (EQ‐5D‐5L), St. George's Respiratory Questionnaire‐COPD (SGRQ‐C), and Beck Depression Inventory (BDI).

### 2.2 Questionnaires

The SGRQ is a disease‐specific quality of life assessment tool that is validated in both asthma and COPD.^8^, ^9^ In the current study, the COPD specific version (SGRQ‐C) was used.^10^ The questionnaire consists of 76 items that are divided into three parts, measuring symptoms, activity limitation, and the social and emotional impact of the disease. Each score ranges from 0 (no impairment) to 100 (maximum perceived distress). Higher scores mean a poorer quality of life.

The CAT is a self‐administered questionnaire that measures health‐related quality of life. The total CAT score was calculated for each individual by summing the points for each variable. CAT scores range from 0 to 40, with a score of 0 indicating no impairment.

Depressive symptoms were assessed using the BDI, a 21‐item self‐report scale for assessing depressive symptoms. Total scores range from 0 to 63. In patients with medical illness, a score of 16 or higher indicates moderate to severe depressive symptoms.^11^

The Fagerstrőm test for nicotine dependence (FTND) and its adapted versions for ECs were used to measure nicotine dependence. The scores from each of the six questions of the FTND were summed and an overall total score for nicotine dependence was calculated. All the questionnaires were administered in‐person to each of the participants.^12^, ^13^

### 2.3 Statistical analysis

Baseline characteristics were given as percentages for categorical variables and as estimated means (± standard deviation) for continuous variables according to the smoking status. We compared clinical characteristics between exclusive ECs users and dual users. Categorical variables and continuous variables were compared using the chi‐square test and Student's *t*‐test, respectively. A two‐sided *p* value <0.05 was considered statistically significant. All statistical analyses were performed using SPSS for Windows software (ver. 20.0; IBM Corp., Armonk, NY, USA).

## 3 RESULTS

### 3.1 Baseline characteristics

Of the 51 participants, 47 (92.2%) were male. The mean age of the participants was 41.8 ± 12.7 years. The 40s group had the highest rate (16, 31.4%) and the 70s group had the lowest rate (1, 2.0%) in the total population. The mean body mass index (BMI) was 25.2 kg/m^2^. In the pulmonary function test, the mean value for predicted forced expiratory volume in 1 s (FEV_1_), forced vital capacity (FVC), and FEV_1_/FVC was 84.8%, 88.3%, and 73.7, respectively. Chronic airway disease included 11 cases of asthma (21.6%), six cases of COPD (11.8%), 39 cases of chronic bronchitis (76.5%), two cases of emphysema (3.9%), one case of bronchiectasis (2.0%), one case of chronic allergic rhinitis (2.0%), and one case of lung cancer (2.0%) (Table 1).

**TABLE 1:** Baseline characteristics of patients with chronic airway disease using e‐cigarettes (*N* = 51)

Variable	Data
Sex, male	47 (92.2)
Age, years	41.8 ± 12.7
Body mass index, kg/m^2^	25.2 ± 3.6
Pulmonary function test	
FEV_1_/FVC	73.8 ± 18.0
FVC % predicted	88.3 ± 22.0
FEV_1_ % predicted	84.8 ± 25.4
Comorbidities	
Asthma	11 (21.6)
COPD	6 (11.8)
Chronic bronchitis	39 (76.5)
Lung emphysema	2 (3.9)
Bronchiectasis	1 (2.0)
Allergic rhinitis	1 (2.0)
Lung cancer	1 (2.0)

*Note*: Data are presented as number (%) or mean ± standard deviation.

Abbreviations: COPD, chronic obstructive pulmonary disease; FEV_1_, forced expiratory volume in 1 s; FVC, forced vital capacity.

### 3.2 The current status of ECs uses

Of the 51 participants, 49 (96.1%) had a history of CCs use. Of these, 30 (61.2%) were currently smoking CCs. The mean pack years (Pys) was 18.3. Thirty‐four (66.7%) participants reported current ECs use and 17 (33.3%) were former ECs users who have used ECs within 1 month. Nineteen (37.3%) were the current dual users (ECs and CCs).

The proportion of using the cigarette type of ECs was higher (33, 64.7%) than those using liquid type of ECs (22, 43.1%). There were four (7.8%) dual users for liquid‐ and cigarette‐type ECs. The mean duration of liquid‐type ECs use was 12.5 months. Of the liquid‐type ECs users, 13 (59.1%) used flavoured e‐liquids. The liquid‐type ECs users smoked 11.2 times a day with 13.4 puff/frequency. In the case of cigarette‐type ECs, the mean duration of ECs use was 23.4 months with 10.8 sticks a day.

After ECs use, 41.2% of CCs users demonstrated reduced consumption of CCs and the amount of reduction was 9.9 cigarettes. At the time of the survey, 39 (76.5%) participants answered to had a plan of smoking cessation, 26 (51.0%) had a plan of smoking cessation in a month, and 33 (64.7%) had a plan of smoking cessation in 6 months. In the past year, 23 (45.1%) participants tried smoking cessation attempts for more than 24 h and five (9.8%) participants used nicotine replacement therapy to quit smoking (Table 2).

**TABLE 2:** Current status of patients with chronic airway disease using e‐cigarettes (*N* = 51)

Variable	Data
Cigarette smoke	49 (96.1)
Current smoker	30 (61.2)
Pack years	18.3 ± 15.6
E‐cigarette smoke	
Current user	34 (66.7)
Ex user (within 1 month)	17 (33.3)
Liquid type	22 (43.1)
Duration (month)	12.6 ± 15.3
Flavours	13 (59.1)
Frequency/day	11.2 ± 8.4
Puff/frequency	13.4 ± 9.2
Cigarette type	33 (64.7)
Duration (month)	23.4 ± 30.1
Stick/day	10.8 ± 6.5
Combination (liquid + cigarette types ECs)	4 (7.8)
Current CCs + ECs	19 (37.3)
Reduction in cigarette after the use of ECs	21 (41.2)
Reduced amounts (cigarettes/day)	10.0 ± 6.5
Attempts to quit smoking	2.6 ± 3.0
Current plan to quit smoke	39 (76.5)
Within 1 month	26 (51)
Within 6 months	33 (64.7)
Quitting smoking for more than 24 h in a year	23 (45.1)
Nicotine replacement	
Yes	5 (9.8)
No	46 (90.2)

*Note*: Data are presented as number (%) or mean ± standard deviation.

Abbreviations: CCs, conventional cigarettes; ECs, e‐cigarettes.

**FIGURE 1:**
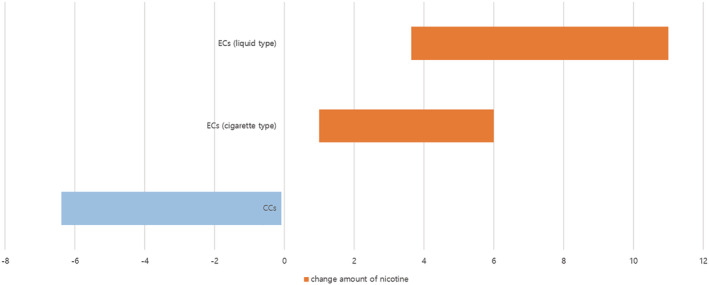
The change of nicotine amounts according to the smoking pattern

After ECs use, the number of cigarettes decreased to an average of 10 cigarettes, but the increased number of ECs was 11 frequency/day for liquid type and 10 for stick type. If inhalation of 10 times of the liquid type of ECs (consuming about 0.04–0.05 g of liquid) is converted to one regular CC, the nicotine content in the smoke of a liquid type of ECs is 0.3–0.7 mg in terms of the amount of one CC, similar to the standard for CCs (0.01–0.7 mg). Also, nicotine content in cigarette type of ECs is 0.1–0.5 mg, which is similar to the content in CCs.^14^ Our results revealed that ECs only change the type of smoking behaviour from cigarettes to ECs without any reduction in the smoke arising from nicotine (Figure 1).

### 3.3 Participants' perception for ECs

We surveyed the participants' perceptions of ECs. Participants reported their views as follows: “ECs are less harmful than CCs” (41, 80.4%), followed by “ECs are just as harmful as CCs” (8, 15.7%), and “ECs are more harmful than CCs” (2, 3.9%). ECs were considered less harmful than CCs because they have few harmful substances (38, 92.7%) or no harmful substances (3, 7.3%).

The number of participants who thought that ECs can help them quit smoking was lower than those who had a controversial opinion (18, 35.3% vs. 33, 64.7%). Thirty‐four (66.7%) participants answered that ECs can cause indirect smoking harm, but 17 (33.4%) answered that ECs do not cause any harm.

**FIGURE 2:**
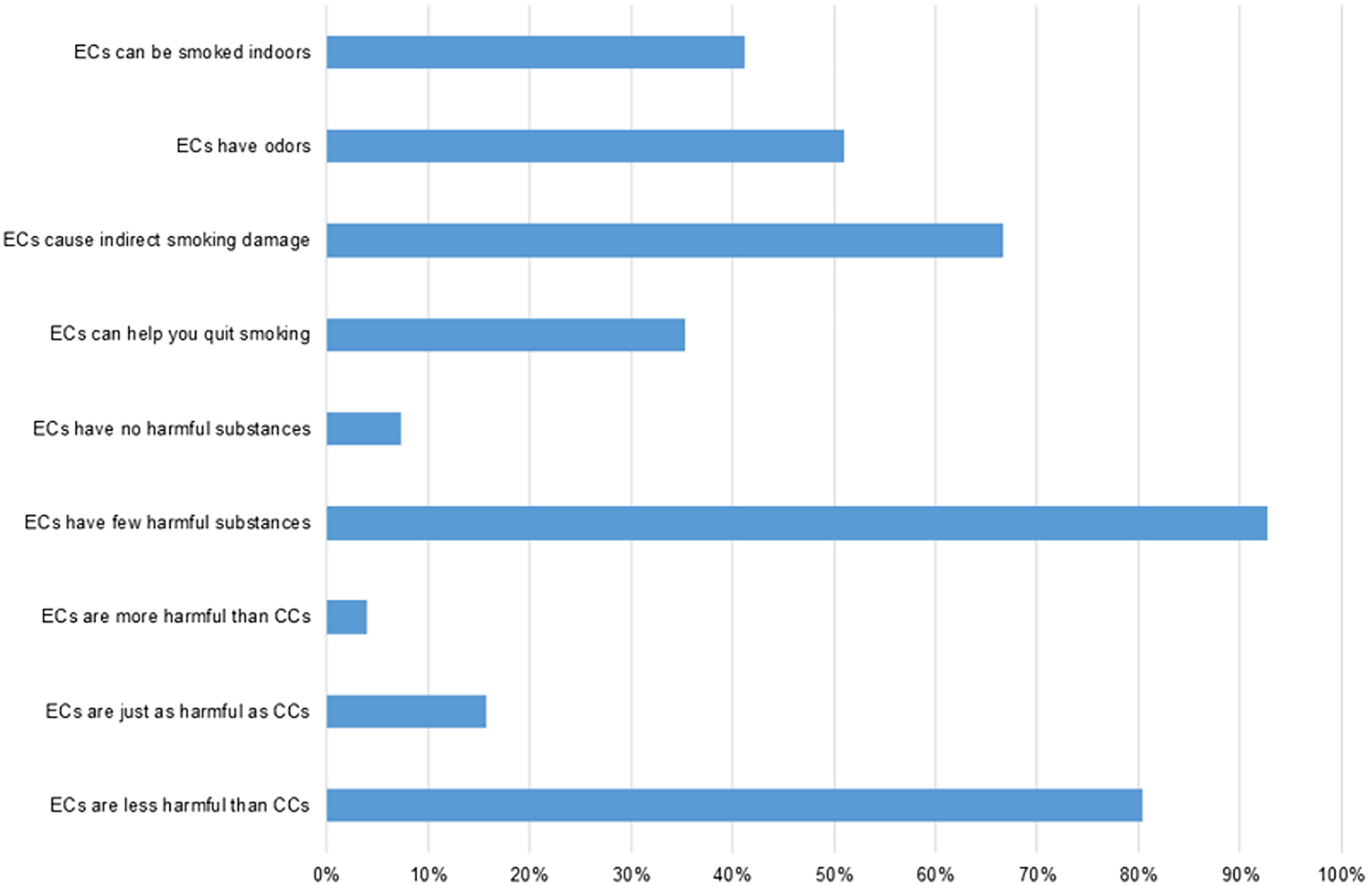
Perception of ECs among users with chronic airway disease or respiratory symptoms

In terms of odour, 26 (51.0%) participants thought that ECs had a smell, whereas 49.0% (*n* = 25) reported that ECs lacked any smell. About half of the participants reported that they could use ECs indoors (41.2%, *n* = 21), and 56.9% reported that they could not use ECs indoors (Figure 2).

### 3.4 The reasons to select ECs by patients with chronic airway disease

Most of the participants (80.4%, *n* = 41) selected ECs because ECs seemed to be less harmful than CCs, followed by “no tobacco odour” (78.4%, *n* = 40), “to help quit smoking” (45.0%, *n* = 23), “to reduce amounts of cigarette smoking” (37.2%, *n* = 19), “easy to smoke indoors” (31.4%, *n* = 16), “good flavour” (21.6%, *n* = 11), “easy to carry and use” (21.6%, *n* = 11), “advice by friends” (15.7%, *n* = 8), “tastes good” (11.7%, *n* = 6), “curiosity” (39.2%, *n* = 2), “looks great” (2.0%, *n* = 1), “cheaper than CCs” (2.0%, *n* = 1), “trendy” (2.0%, *n* = 1), and “easy to get” (2.0%, *n* = 1).

### 3.5 Attitudes, intentions, and restrictions related to ECs use

Table 3 shows the data regarding the attitudes and intentions to use ECs. The majority of the participants thought that ECs have fewer health risks than CCs (58.8%) but answered that ECs should be allowed where smoking is prohibited (13.7%); ECs have been shown to help smokers quit smoking (15.7%) and to reduce cigarette consumption (33.3%). Only 7.8% of the participants believed that the Food and Drug Administration (FDA) should regulate the use of ECs.

**TABLE 3:** The attitude towards ECs and intention to use (*N* = 51)

Variable	Number (%)
Attitude towards ECs (multiple choices)	
ECs have fewer health risks in comparison to regular cigarettes	30 (58.8)
You should be able to use ECs in places where smoking is prohibited	7 (13.7)
ECs have been shown to help smokers quit smoking	8 (15.7)
ECs help people cult down on cigarettes or quit smoking	17 (33.3)
The FDA should regulate the use of ECs	4 (7.8)
Intention to use (multiple choices)	
I plan to eventually quit the use of ECs and CCs	19 (37.3)
I plan to use them as a complete replacement for CCs	14 (27.5)
I plan to use them when I cannot use cigarettes	12 (23.5)
I plan to use them as a partial replacement for CCs	6 (11.8)
I plan to gradually switch from CCs to ECs	5 (9.8)
I plan to use them in addition to CCs	4 (7.8)
I plan to just experiment with it—I have not yet made up my mind	3 (5.9)
I just wanted to try something different and new	3 (5.9)

Abbreviations: CCs, conventional cigarettes; ECs, e‐cigarettes; FDA, Food and Drug Administration.

Most of the participants reported that they planned to eventually quit the use of ECs and CCs (37.3%). Fewer participants reported that they were planning to use them as a complete replacement for CCs (27.5%) or they were planning to use ECs where they cannot use CCs (23.5%) or switching gradually from CCs to ECs (9.8%).

### 3.6 Respiratory symptoms and assessment of the quality of life

We surveyed the respiratory symptoms in EC users using questionnaires. About 70% of the EC users complained of cough and sputum. Fewer participants (11, 21.6%) reported that they had chest pain. Of these, half of the participants answered that they had cough or sputum for more than 1 month. Also, most of the participants had no dyspnoea, but about 20% of participants had mild dyspnoea. EQ‐5D‐5L, which represents the participants' quality of life, was 0.9 points, and the visual analogue scale rating was 70.9.

### 3.7 Comparison of clinical characteristics between exclusive EC users and dual users

We further compared the clinical characteristics of dual users and exclusive EC users. Age, BMI, total PYs of cigarettes, EQ‐5D‐5L index, pulmonary function test, comorbidities, and plan for smoking cessation were not different between the two groups. The total SGRQ was not different between the groups, but symptom SGRQ (42.2 ± 17.4 vs. 30.6 ± 17.40, *p* = 0.05) was higher in dual users. The mean BDI score (9.4 ± 8.2 vs. 4.2 ± 4.5, *p* = 0.016) and the proportion of cases with moderate to severe depressive symptoms (30.4% vs. 4.8%, *p* = 0.027) were higher in dual users than exclusive EC users (Table 4).

**TABLE 4:** Comparison of symptoms and lung functions between exclusive EC users and dual users

Variable	Exclusive ECs users (*n* = 29)	Dual users (*n* = 19)	*p* value
Age, years	42.7 ± 10.4	41.1 ± 14.3	0.643
EQ‐5D‐5L, index	0.9 ± 0.1	0.9 ± 0.1	0.333
Total SGRQ	7.5 ± 6.8	13.5 ± 12.2	0.091
Symptom SGRQ	30.6 ± 17.4	42.2 ± 17.4	0.049
Activity SGRQ	1.7 ± 4.2	10.0 ± 20.1	0.093
Impact SGRQ	3.9 ± 6.6	5.8 ± 10.6	0.515
BDI score, mean	4.2 ± 4.5	9.4 ± 8.2	0.016
High BDI score ≥16	1 (3.4)	7 (36.8)	0.027
Reduction in cigarette amount after EC use	11.9 ± 6.6	7.8 ± 6.1	0.174
PFT (post BD)			
FVC, %	94.6 ± 18.2	89.5 ± 14.7	0.394
FEV_1_, %	94.4 ± 20.6	87.1 ± 19.3	0.322
FEV_1_/FVC	77.7 ± 11.5	78.4 ± 12.7	0.877

*Note*: Data are presented as mean ± standard deviation or number (%).

Abbreviations: BMI, body mass index; BDI, Beck Depression Inventory; EC, e‐cigarettes; EQ‐5D‐5L, EuroQol‐5 dimension‐5 level; FEV_1_, forced expiratory volume in 1 s; FVC, forced vital capacity; PFT, pulmonary function test; SGRQ, St. George's Respiratory Questionnaire.

The average FTND score among the ECs users was 3.7 ± 2.9. The average FTND score among dual users was over twice as high compared with exclusive ECs users (mean 4.6 vs. 2.4, *p* = 0.006). Dual users were more likely to be difficult to refrain from the use of ECs in places where it was forbidden (50.0% vs. 4.8%, *p* = 0.001) and use ECs more frequently during the first hours after waking than during the rest of the day (46.4% vs. 9.5%, *p* = 0.006) (Table 5).

**TABLE 5:** Comparison of Fagerstrőm test for nicotine dependence between exclusive ECs users and dual users

Questions	Exclusive ECs users	Dual users	*p* value
How soon after waking up do you reach for an ECs?			
Within 30 min	10 (34.4)	12 (63.2)	0.191
Do you find it difficult to refrain from the use of ECs in places where it is forbidden?			
Yes	1 (4.8)	14 (50.0)	0.001
Which ECs would you hate most to give up?			
The first one in the morning	2 (9.5)	6 (21.4)	0.265
How many times a day do you use ECs? (Number of e‐smoking sessions: one e‐smoking session consist of 15 puffs or approximately 10 min of use)			
10 or less	8 (38.1)	8 (28.6)	0.337
11 to 20	13 (61.9)	16 (57.1)	
21–30	0 (0.0)	2 (7.1)	
31 or more	0 (0.0)	2 (7.1)	
Do you use ECs more frequently during the first hours after waking than during the rest of the day?			
Yes	2 (9.5)	13 (46.4)	0.006
Do you use ECs if you are so ill that you are in bed most of the day?			
Yes	5 (23.8)	13 (46.4)	0.104
FTND summary score			
FTND (mean ± SD)	2.4 ± 1.8	4.6 ± 3.3	0.006

*Note*: Data are presented as number (%).

Abbreviations: ECs, e‐cigarettes; FTND, Fagerstrom test for nicotine dependence.

## 4 DISCUSSION

We investigated the clinical characteristics of adult EC users with chronic airway disease or chronic respiratory symptoms. Most of the participants were male, and dominant airway diseases were asthma and COPD. Most of the subjects had a history of CCs smoking, and 19 were dual users for current CCs and ECs. Most of the subjects started ECs use due to health‐related problems (harm reduction, no foul odour, or for cigarette smoking cessation). A comparison between exclusive ECs users and dual users revealed higher symptom SGRQ, the proportion of cases with moderate to severe depressive symptoms, and average FTND scores for ECs in dual users than exclusive ECs users, respectively.

In our study, about 35% of participants believed that ECs would help smokers quit smoking, and a quarter of participants intended to use ECs as a complete replacement for CCs. Previous research suggests that ECs have the potential to assist smokers to quit or reduce smoking. ECs deliver nicotine into the bloodstream and reduces tobacco withdrawal in a manner effective as nicotine replacement therapy.^15^, ^16^ In a randomized controlled trial (RCT) that investigated the effectiveness of ECs versus nicotine patches for smoking cessation, abstinence rates at 6 months were 7.3% with ECs and 5.8% with nicotine patches and 4.1% with placebo ECs. Although the abstinence rate was higher in ECs users, the differences did not demonstrate a statistical significance.^4^ However, abstinence rates reported in the reported RCT were considerably lower in comparison with the abstinence rate of varenicline, which is widely used as a smoking cessation therapy. Continuous 1‐year abstinence rate of varenicline ranged from 22% to 35%.^17^ In a meta‐analysis that included 38 studies, ECs use was not associated with quitting compared with studies on only smokers interested in cigarette cessation.^6^

ECs generate less tar and carcinogens than conventional cigarettes; therefore, ECs users believe that its use may reduce disease caused by harmful components.^18^ In our study, the majority of the participants answered that ECs have fewer health risks and are less harmful in comparison with regular cigarettes. The health effects of ECs are controversial. There is no doubt that the concentration of ingredients in ECs' aerosol is lower than that of conventional cigarettes.^19^ The main ingredients in ECs liquids are the compounds that are used in pharmaceutical, cosmetic products, and food. However, there is a growing body of evidence on the association between ECs and acute lung injury. A total of 2807 electronic cigarettes or vaping product use‐associated lung injury (EVALI) cases or deaths have been reported to the Center of Disease Control and Prevention (CDC) as of 18 February 2020.^20^ The FDA identified that vitamin E acetate is associated with EVALI. When inhaled, vitamin E acetate is incorporated into the phospholipids that are composed of surfactant, thereby increasing its permeability and resulting in function deterioration.^21^

In the case of chronic airway disease, ECs use has been reported to be common in adults with or at risk for COPD and was associated with worse pulmonary‐related health outcomes including lung function decline and exacerbations.^3^ It has been reported that ECs use is positively related to asthma and is associated with an increased risk of chronic bronchitis symptoms, such as chronic cough and phlegm in adolescents.^22^ These findings could be explained based on the theory that ECs vapour causes inhibition of cough reflex sensitivity.^23^ Chronic exposure to ECs vapour for 4 months did not induce pulmonary inflammation or emphysema but altered lung lipid homeostasis in alveolar macrophage and epithelial cells independent of the presence of nicotine in a murine model.^24^ Also, exposure to inhaled nicotine‐containing ECs fluid triggered the effects associated with the development of COPD including cytokine expression, airway hyperreactivity, and lung tissue destruction.^25^

The important finding of the current study was that nicotine dependence levels measured with FTND were higher in dual users than exclusive ECs users in adults with chronic airway disease or chronic respiratory symptoms. Also, ECs use increased proportionally to the reduction in the number of cigarettes for liquid type to 11 times and stick type to 10 sticks, which is approximately the same amount of nicotine in the Korean products. In line with this finding, a recent study reported that ECs users are less dependent on nicotine than current cigarette smokers.^26^, ^27^ Among healthy smokers, dual users consumed significantly fewer cigarettes but demonstrated no difference in cotinine levels, thereby suggesting that they supplemented their nicotine requirement by using ECs.^28^ Dual adult users with or at risk for COPD had higher nicotine dependence than those who smoked only CCs.^8^ Multiple tobacco product users were associated with past quit attempts. Smokers with prior failed quitting may turn to other tobacco products to cut down on their cigarette consumption without complete cessation.^29^ A previous study demonstrated no significant decrease in cotinine levels from baseline to 1 or 2 months in regular smokers who initiated the use of ECs.^30^ Also, some ECs users can obtain a large amount of nicotine from ECs similar or higher levels observed in cigarette smokers.^31^ Higher levels of nicotine levels in dual users than exclusive users could be explained based on the concept that dual users cut some extent of their cigarette consumption after they start using ECs, but they supplement their nicotine through ECs use.

Cigarette smoking is known to be associated with higher levels of depressive symptoms and impairment of quality of life.^32^ Also, the ECs use is known to be associated with a history of clinical diagnosis of depression than never users.^33^ In a previous study, dual users had higher proportions of continuous depressive mood that lasted for > 2 weeks than cigarette only smokers. Park et al. showed that smokers with high psychological distress levels tended to be dual users than exclusive ECs users or exclusive cigarette users.^34^ In line with the previous studies, our results indicate that dual users had a higher mean BDI score and the proportion of participants with a BDI score of 16 or higher, which means moderate to severe depressive symptoms than exclusive ECs users. In Korea, the smoking pattern has changed from only users to dual and triple users.^7^ Therefore, cessation treatment and raising the importance of health concerns in case of dual users are needed.

This pilot has several limitations worth noting. First, a large number of subjects could not be recruited due to the relatively short registration period. Our study was too small to allow reliable comparisons between subgroups of patients. Further research is needed through large‐scale recruitment in the future. Second, the inference of a causal relationship between ECs use and bronchitis symptoms, nicotine dependence, and depressive symptoms was limited because the cross‐sectional nature of our study did not allow us to clarify the direction of the association. Because our study aimed to investigate the current status of ECs use patterns in patients with chronic airway disease and ECs users were registered, there was no need to include a control group to evaluate the influence of ECs.

## 5 CONCLUSIONS

In conclusion, most of the ECs users had a history of CCs smoking and dual users for current CCs and ECs. Most of the subjects started ECs use due to health‐related reasons and considered ECs as less harmful and intended to quit or reduce CCs use. But the use of ECs did not help in reducing the amount of nicotine. Dual users had higher symptom SGRQ, the proportion of cases with moderate to severe depressive symptoms, and average FTND scores for ECs than exclusive ECs users. One hypothesis that comes from these results is that greater nicotine dependence may influence behaviours, habits, and views about ECs. In the future, large‐scale studies are needed to obtain more concrete evidence about the smoking behaviours and nicotine dependence according to ECs use in patients with chronic airway disease.

## CONFLICT OF INTEREST

The authors declare that they have no competing interests.

## ETHICS STATEMENT

The survey was performed in accordance with the Declaration of Helsinki 7th version, and written informed consent was obtained from all the participants before completing the survey. The present study was approved by the Institutional Review Board (IRB) of the Kyung Hee University College of Medicine (IRB number: KHUH 2019‐10‐033‐004).

## AUTHOR CONTRIBUTIONS

HSK, JWJ, JYK, and HSC contributed to protocol development, data analysis, and drafted the manuscript. HJP, DIP, and JSP contributed to study conception and participated in its coordination. JHP, SHL, and CEM contributed to data checking and information retrieval. HSK, JWJ, HJP, JYK, and HSC designed the study and contributed to the overall management of the study. All authors reviewed the manuscript and approved the final version of the manuscript.

## Data Availability

The data that support the findings of this study are available from the corresponding author upon reasonable request.

## References

[1] Nayir E , Karacabey B , Kirca O , Ozdogan M . Electronic cigarette (e‐cigarette). J Oncol Sci. 2016;2(1):16‐20.

[2] Lewek P , Wozniak B , Maludzinska P , Smigielski J , Kardas P . E‐cigarette use and its predictors: results from an online cross‐sectional survey in Poland. Tob Induc Dis. 2019;17:79. 31772557 10.18332/tid/113093 PMC6856825

[3] Bowler RP , Hansel NN , Jacobson S , et al. Electronic cigarette use in US adults at risk for or with COPD: analysis from two observational cohorts. J Gen Intern Med. 2017;32(12):1315‐1322. 28884423 10.1007/s11606-017-4150-7 PMC5698219

[4] Bullen C , Howe C , Laugesen M , et al. Electronic cigarettes for smoking cessation: a randomised controlled trial. Lancet. 2013;382(9905):1629‐1637. 24029165 10.1016/S0140-6736(13)61842-5

[5] Ghosh S , Drummond MB . Electronic cigarettes as smoking cessation tool: are we there? Curr Opin Pulm Med. 2017;23(2):111‐116. 27906858 10.1097/MCP.0000000000000348 PMC5480094

[6] Kalkhoran S , Glantz SA . E‐cigarettes and smoking cessation in real‐world and clinical settings: a systematic review and meta‐analysis. Lancet Respir Med. 2016;4(2):116‐128. 26776875 10.1016/S2213-2600(15)00521-4 PMC4752870

[7] Korea Disease Control and Prevention Agency . E‐cigarette smoking is not helpful for smoking cessation and health. https://www.cdc.go.kr/board.es?mid=a20501000000%26bid=0015%26act=view%26list_no=366799#

[8] Alter P , Mayerhofer BA , Kahnert K , et al. Prevalence of cardiac comorbidities, and their underdetection and contribution to exertional symptoms in COPD: results from the COSYCONET cohort. Int J Chron Obstruct Pulmon Dis. 2019;14:2163‐2172. 31571852 10.2147/COPD.S209343 PMC6759215

[9] Mullerova H , Cockle SM , Gunsoy NB , Nelsen LM , Albers FC . Clinical characteristics and burden of illness among adolescent and adult patients with severe asthma by asthma control: the IDEAL study. J Asthma. 2020;58(4):1‐12. 10.1080/02770903.2019.1708095 31874051

[10] Paap MC , Lange L , van der Palen J , Bode C . Using the Three‐Step Test Interview to understand how patients perceive the St. George's Respiratory Questionnaire for COPD patients (SGRQ‐C). Qual Life Res. 2016;25(6):1561‐1570. 26615616 10.1007/s11136-015-1192-3 PMC4870291

[11] Steer RA , Clark DA , Beck AT , Ranieri WF . Common and specific dimensions of self‐reported anxiety and depression: the BDI‐II versus the BDI‐IA. Behav Res Ther. 1999;37(2):183‐190. 9990749 10.1016/s0005-7967(98)00087-4

[12] Jankowski M , Krzystanek M , Zejda JE , et al. E‐cigarettes are more addictive than traditional cigarettes—a study in highly educated young people. Int J Environ Res Public Health. 2019;16(13):2279. 10.3390/ijerph16132279 PMC6651627 31252671

[13] Piper ME , Baker TB , Benowitz NL , Smith SS , Jorenby DE . E‐cigarette dependence measures in dual users: reliability and relations with dependence criteria and e‐cigarette cessation. Nicotine Tob Res. 2020;22(5):756‐763. 30874804 10.1093/ntr/ntz040 PMC7368344

[14] Ministry of Food and Drug Safety (Republic of Korea) . Analysis of harmful ingredients in conventional cigarette and e‐cigarettes. https://www.mfds.go.kr/brd/m_99/view.do?seq=36783

[15] Bullen C , McRobbie H , Thornley S , Glover M , Lin R , Laugesen M . Effect of an electronic nicotine delivery device (e‐cigarette) on desire to smoke and withdrawal, user preferences and nicotine delivery: randomised cross‐over trial. Tob Control. 2010;19(2):98‐103. 20378585 10.1136/tc.2009.031567

[16] Vansickel AR , Eissenberg T . Electronic cigarettes: effective nicotine delivery after acute administration. Nicotine Tob Res. 2013;15(1):267‐270. 22311962 10.1093/ntr/ntr316 PMC3524053

[17] Garrison GD , Dugan SE . Varenicline: a first‐line treatment option for smoking cessation. Clin Ther. 2009;31(3):463‐491. 19393839 10.1016/j.clinthera.2009.03.021

[18] Farsalinos K . Electronic cigarettes: an aid in smoking cessation, or a new health hazard? Ther Adv Respir Dis. 2018;12:1753465817744960. 29214890 10.1177/1753465817744960 PMC5937152

[19] Thirion‐Romero I , Perez‐Padilla R , Zabert G , Barrientos‐Gutierrez I . Respiratory impact of electronic cigarettes and “low‐risk” tobacco. Rev Invest Clin. 2019;71(1):17‐27. 30810544 10.24875/RIC.18002616

[20] Centers for Disease Control and Prevention . Centers for Disease Control and Prevention. Outbreak of Lung Injury Associated with E‐cigarette Use, or Vaping. https://www.cdc.gov/tobacco/basic_information/e-cigarettes/severe-lung-disease.html#latest-information

[21] Winnicka L , Shenoy MA . EVALI and the pulmonary toxicity of electronic cigarettes: a review. J Gen Intern Med. 2020;35(7):2130‐2135. 32246394 10.1007/s11606-020-05813-2 PMC7351931

[22] McConnell R , Barrington‐Trimis JL , Wang K , et al. Electronic cigarette use and respiratory symptoms in adolescents. Am J Respir Crit Care Med. 2017;195(8):1043‐1049. 27806211 10.1164/rccm.201604-0804OC PMC5422647

[23] Dicpinigaitis PV . Effect of tobacco and electronic cigarette use on cough reflex sensitivity. Pulm Pharmacol Ther. 2017;47:45‐48. 28185897 10.1016/j.pupt.2017.01.013

[24] Madison MC , Landers CT , Gu BH , et al. Electronic cigarettes disrupt lung lipid homeostasis and innate immunity independent of nicotine. J Clin Invest. 2019;129(10):4290‐4304. 31483291 10.1172/JCI128531 PMC6763255

[25] Garcia‐Arcos I , Geraghty P , Baumlin N , et al. Chronic electronic cigarette exposure in mice induces features of COPD in a nicotine‐dependent manner. Thorax. 2016;71(12):1119‐1129. 27558745 10.1136/thoraxjnl-2015-208039 PMC5136722

[26] Gonzalez Roz A , Secades Villa R , Weidberg S . Evaluating nicotine dependence levels in e‐cigarette users. Adicciones. 2017;29(2):136‐138. 28170058 10.20882/adicciones.905

[27] Rhoades DA , Comiford AL , Dvorak JD , et al. Vaping patterns, nicotine dependence and reasons for vaping among American Indian dual users of cigarettes and electronic cigarettes. BMC Public Health. 2019;19(1):1211. 31477072 10.1186/s12889-019-7523-5 PMC6721166

[28] Piper ME , Baker TB , Benowitz NL , Kobinsky KH , Jorenby DE . Dual users compared to smokers: demographics, dependence, and biomarkers. Nicotine Tob Res. 2019;21(9):1279‐1284. 30365010 10.1093/ntr/nty231 PMC7182769

[29] Lee YO , Hebert CJ , Nonnemaker JM , Kim AE . Multiple tobacco product use among adults in the United States: cigarettes, cigars, electronic cigarettes, hookah, smokeless tobacco, and snus. Prev Med. 2014;62:14‐19. 24440684 10.1016/j.ypmed.2014.01.014

[30] Berg CJ , Barr DB , Stratton E , Escoffery C , Kegler M . Attitudes toward e‐cigarettes, reasons for initiating e‐cigarette use, and changes in smoking behavior after initiation: a pilot longitudinal study of regular cigarette smokers. Open J Prev Med. 2014;4(10):789‐800. 25621193 10.4236/ojpm.2014.410089 PMC4304080

[31] Etter JF . Levels of saliva cotinine in electronic cigarette users. Addiction. 2014;109(5):825‐829. 24401004 10.1111/add.12475

[32] Milic M , Gazibara T , Pekmezovic T , et al. Tobacco smoking and health‐related quality of life among university students: mediating effect of depression. PLoS One. 2020;15(1):e0227042. 31914158 10.1371/journal.pone.0227042 PMC6948726

[33] Obisesan OH , Mirbolouk M , Osei AD , et al. Association between e‐cigarette use and depression in the Behavioral Risk Factor Surveillance System, 2016–2017. JAMA Netw Open. 2019;2(12):e1916800. 31800073 10.1001/jamanetworkopen.2019.16800 PMC6902792

[34] Park SH , Lee L , Shearston JA , Weitzman M . Patterns of electronic cigarette use and level of psychological distress. PLoS One. 2017;12(3):e0173625. 28278239 10.1371/journal.pone.0173625 PMC5344459

